# Migraines and the association of cognitive impairment: a one- and two-sample mendelian randomization analysis

**DOI:** 10.1080/19585969.2026.2636459

**Published:** 2026-03-09

**Authors:** Chyi-Huey Bai, Hsien-Yu Fan, Hui-An Lin, Sheng-Feng Lin

**Affiliations:** a Department of Public Health, School of Medicine, College of Medicine, Taipei Medical University, Taipei, Taiwan; b School of Public Health, College of Public Health, Taipei Medical University, Taipei, Taiwan; c Nutrition Research Center, Taipei Medical University Hospital, Taipei, Taiwan; d School of Nutrition and Health Sciences, College of Nutrition, Taipei Medical University, Taipei, Taiwan; e Department of Emergency Medicine, Taipei Medical University Hospital, Taipei, Taiwan; f Department of Emergency Medicine, School of Medicine, College of Medicine, Taipei Medical University, Taipei, Taiwan; g Department of Evidence-Based Medicine, Taipei Medical University Hospital, Taipei, Taiwan

**Keywords:** Cognitive impairment, mendelian randomisation, migraine

## Abstract

**Background:**

Cognitive impairment is widely reported in migraineurs. A Mendelian randomisation (MR) approach, similar to a randomised-controlled trial, employs single-nucleotide polymorphisms (SNPs) to investigate causal relationships.

**Methods:**

This study comprised one- and two-sample MR analyses of the Taiwan Biobank. Three strategies were used to obtain causal estimates: (1) a polygenic risk score (PRS) method—several SNPs associated with migraines were constructed as a single instrument variable; (2) a meta-analysis of genome-wide association study (GWAS) statistics for traits of migraines and cognitive impairment in the framework of a one-sample MR; and (3) a two-sample MR analysis with a meta-analysis of GWAS statistics in two distinct datasets (IEU GWAS database and the Taiwan Biobank).

**Results:**

In strategy 1, the PRS constructed by 18 selected SNPs exhibited a causal association with cognitive impairment (β = −2.31, 95% confidence interval [CI]: −4.56 to −0.06). In strategy 2, a one-sample MR showed migraines were causally associated with cognitive impairment (inverse-variance weighted [IVW] estimator β = 2.90; 95% CI: 0.90–4.89). In strategy 3, a two-sample MR validated migraines to be causally associated with cognitive impairment (IVW estimator β = 2.43; 95% CI: 1.08–3.78).

**Conclusions:**

Migraine, a polygenic disorder, is causally associated with cognitive impairment.

## Graphical Abstract

**Figure:**
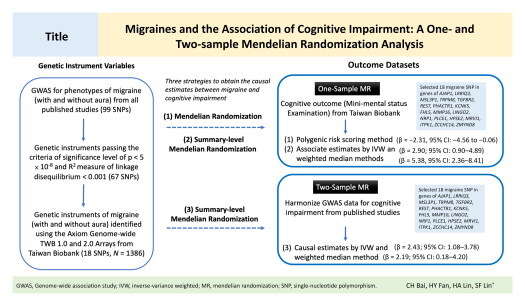

## Introduction

Cognitive impairment is a widely reported symptom in migraineurs. Previous registry studies, however, showed inconsistent results regarding the causal association between cognitive impairment and migraines (Chuang et al. 2013; Wen et al. 2016; Islamoska et al. 2020). Some studies found impaired cognitive performance for migraineurs during interictal (Camarda et al. 2007) and ictal periods (Gil-Gouveia et al. 2016). A recent meta-analysis study (Gu et al. 2022) demonstrated that migraineurs had significant cognitive impairment, especially deficits in the language domain, and migraineurs exhibited a higher risk of all-cause dementia. For the Han Chinese population, observational studies showed that migraineurs also had lower cognitive performance than healthy controls (Chuang et al. 2013; Wang et al. 2016; Wen et al. 2016; Liu et al. 2017). Even more, migraineurs treated with traditional Chinese medicine (Liu et al. 2017) or acupuncture (Huang et al. 2022) exhibited a reduced risk of dementia. In those studies, however, determining a true causal relationship was limited by the cross-sectional nature of the study, small sample sizes (Wang et al. 2016; Wen et al. 2016), and confounding factors (Chuang et al. 2013; Wen et al. 2016; Gu et al., 2022; Liu et al., 2017; Islamoska et al. 2020; Huang et al. 2022). Conversely, a few studies showed no significant difference in cognitive function (Jelicic et al. 2000; Gaist et al. 2005; Wen et al. 2016; Baschi et al. 2019) and dementia occurrence (Martins et al. 2020) between migraineurs and non-migraineurs. However, these studies were limited by the use of non-standardized instruments to measure cognitive function (Gaist et al. 2005), single domain results (Jelicic et al. 2000; Baschi et al. 2019), varied study population from hospital or community settings (Wen et al. 2016), and high attrition rates (Martins et al. 2020). In addition, none of these studies included the Han Chinese population.

Mendelian randomisation (MR), similar to a randomised controlled trial, relies on a random assortment of genetic variants during meiosis to generate a random distribution of genetic variants in a population (Burgess et al. 2016; CRP CHD Genetics Collaboration 2011). This approach aids in estimating a causal relationship between a phenotype and an outcome using genetic variants as instrumental variables (IVs) (CRP CHD Genetics Collaboration 2011). The use of genetic variants as IVs to investigate a causal relationship is based on three assumptions:(Burgess et al. 2016; CRP CHD Genetics Collaboration, 2011) first, genetic IVs are significantly associated with the phenotype (the relevance assumption); second, genetic IVs are significantly associated with an outcome through only the studied phenotype (the exclusion restriction assumption); and third, genetic IVs are independent of other factors that affect the outcome (the independence of IVs assumption) (Labrecque and Swanson 2018). From a genome-wide association study (GWAS), the direction of causality from each genetic variant to the phenotype is disclosed, i.e., by single-nucleotide polymorphisms (SNPs) to an observable trait. With an increasing availability of biobank data that contain both genetic and phenotypic information, there has been a growing trend to use MR to investigate causal relationships.

Initially, most MR studies were conducted in the form of a one-sample MR, namely, both the phenotype and outcome data were collected in the same population. In general, F-statistic values for associations of genetic variants with the phenotype indicate the instrumental strength, i.e., a higher F-statistic value indicates more-robust genetic IVs and stronger statistical power (CRP CHD Genetics Collaboration 2011). In a one-sample MR, using weak IVs can bias the estimates towards confounded results, which usually underestimate the true causal effect. In the last decade, there has been an increasing trend for MR studies conducted in the form of two-sample MRs (Lawlor 2016), namely phenotype and outcome data are collected in two distinct populations, to raise the power and avoid weak instrument bias. Recently, the two-sample MR method was validated for application to a single large dataset (referred to as application of ‘two-sample MR method’ to a ‘one-sample MR population’) (Minelli et al. 2021). In this method, SNPs are selected according to published GWASs, and a single IV is constructed using the polygenic risk scores (PRSs) of selected SNPs.

Located at East Asia, Taiwan has a population of 23 million, among which more than 99% are of the Han Chinese ethnicity. The Taiwan Biobank is a large-scale biomedical database of Han Chinese ancestry. It aimed to enrol 300,000 adult residents of Taiwan (Fan et al. 2008; Chen et al. 2016; Wei et al. 2021) and includes more than 200,000 people. The study is the first large-scale study investigating the association between migraines and cognitive impairment in a Han Chinese population through an MR approach. To determine whether migraines are causally associated with cognitive impairment in Asians, in the present study, we employed MR analyses of the Taiwan Biobank.

## Materials and methods

### Study design

**Figure 1.**
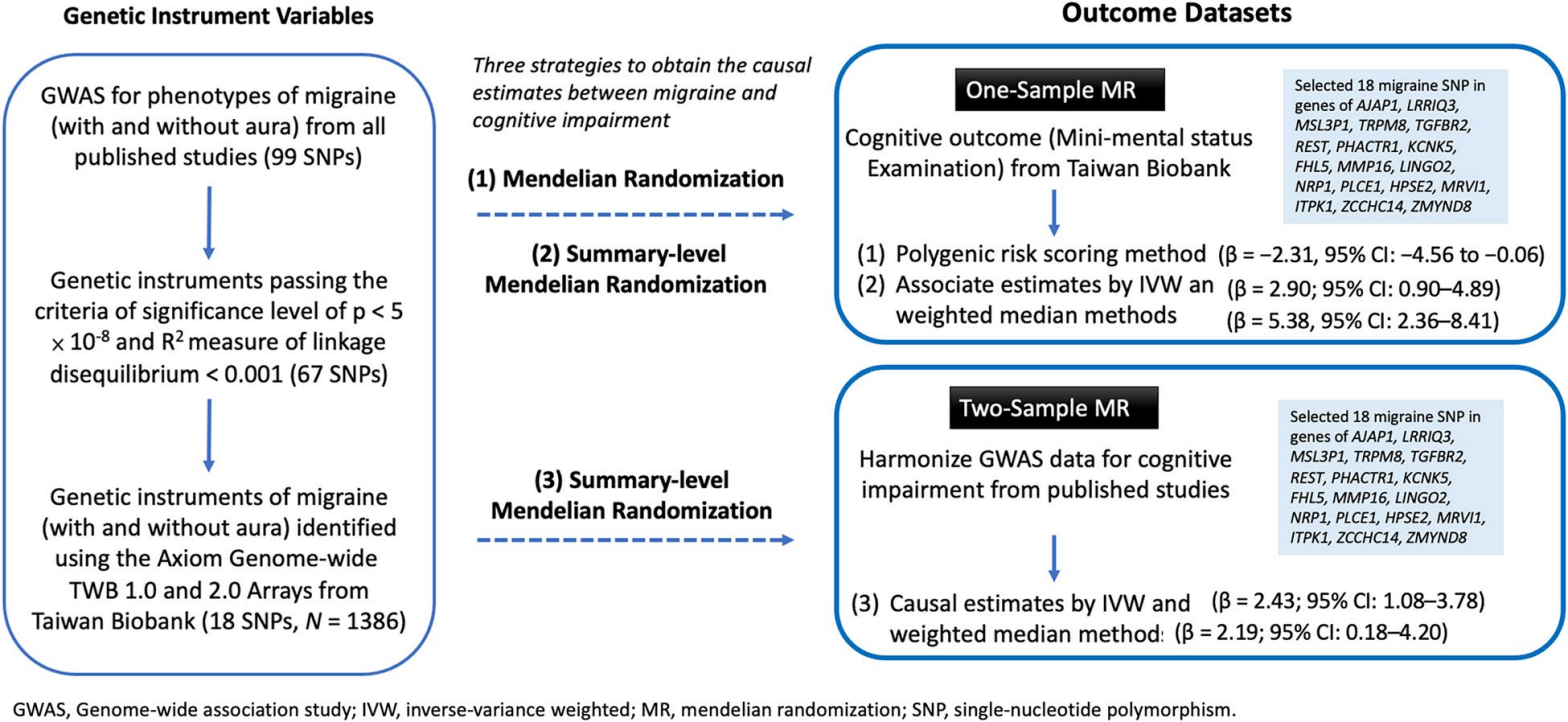
Flow diagram and design of the study.

This study comprised one- and two-sample MR analyses of a single large dataset, namely, application of MR methods to primary and summary association results in overlapping (one-sample) and non-overlapping (two-sample) sets of phenotype and outcome populations, respectively. We employed three strategies to obtain causal estimates between migraines and cognitive impairment: (Islamoska et al. 2020) Strategy 1: a PRS method—a single variable that was constructed by several SNPs associated with migraines in the framework of a one-sample MR (i.e., use of both migraine and cognitive data in the Taiwan Biobank); (Chuang et al. 2013) Strategy 2: a summary level of GWAS data for traits of migraines and cognition in the framework of a one-sample MR (i.e., using identified SNPs of migraines from published large-scale GWAS research;(Anttila et al. 2010; Ligthart et al. 2011; Chasman et al. 2011; Freilinger et al. 2012; Anttila et al. 2013; Pickrell et al. 2016; Gormley et al. 2016; Chen et al. 2018) however, SNP associations with both migraines and cognition were obtained from the Taiwan Biobank dataset); and (Wen et al. 2016) Strategy 3: a summary level of GWAS data in the framework of a two-sample MR (i.e., use of GWAS data for traits of migraines in an open database, and for traits of cognition in the Taiwan Biobank). A flow diagram of the three strategies is summarised in Figure 1.

### Selection of genetic variants (the phenotype dataset)

For all strategies, we identified SNPs associated with migraines using the IEU open GWAS database (gwas.mrcieu.ac.uk). SNPs were selected based on a *p* value of < 5 × 10^−8^ for significance level associated with the trait of migraines. To ensure the independence of the selected SNPs, we applied linkage disequilibrium (LD) clumping using the ‘clump_data’ function from the TwoSampleMR package in R (version 0.6.6), with LD clumping parameters set to a window size of 10,000 kilobases, an r-squared threshold (*R*^2^) of < 0.001, a *p* value thresholds for both index and secondary SNPs of < 1 × 10^−5^. The selected genetic variants (SNPs) were all validated based on seven published large-scale GWAS(22–28) involving participants of European ancestry and one GWAS(29) conducted in a Han Chinese population. For GWAS research (Anttila et al. 2010; Chasman et al. 2011; Ligthart et al. 2011; Freilinger et al. 2012; Anttila et al. 2013; Gormley et al. 2016; Pickrell et al. 2016; Chen et al. 2018) that was analysed in the study, we summarised detailed information regarding the source of the population, sample size, quality control of samples and SNPs, and criteria used for diagnosing migraines in Table 1.

**Table 1.** The selected single-nucleotide polymorphism genome-wide association studies (GWAS).

Study authors	Sample size	Data source	Quality Control	Criteria of diagnosis	SNP (mapped loci)
Antilla et al. (PubMed ID: 20802479) (Anttila et al. 2010)	2,748 migraine, 2,148 migraine with aura and without aura, 589 migraine with aura only, 17 migraine without aura	European descent, including the initial study with three datasets of Finnish cohort, German cohort, Dutch cohort and the replication study with four datasets of Danish cohort, Dutch cohort, Icelandic cohort, and German cohort	Samples were excluded with call rates <97%, non-comparable ancestry, cryptic relatedness a large number of samples, and non-cryptic relatedness of π_hat >12.5% SNPs were excluded with a MAF of <1%, HWE *p* value <10^−6^	ICHD, 2nd edition except German replication cohort (self-reported)	rs1835740 (Intergenic between *MTDH/AEG-1* and *PGCP*), rs12084862 (*SMYD3*), rs17528324 (*INSIG2*), rs17862920 (*TRPM8*), rs2038761 (*MYLK4*), rs6456880 (*ZNF311*), rs2042600 (*NAV2*), rs3794331 (*COG3*), rs10888075 (*SGCZ*), rs473422 (*AQP9*) Note: This study selected the SNPs with the significant association with p-value ≤ 5 × 10^−5^.
Ligthart et al. (PubMed ID: 21448238) (Ligthart et al. 2011)	2,446 migraine, 8,534 controls	European descent, including population-based studies of Age, Gene/Environment Susceptibility – Reykjavik Study (AGES-RS), Erasmus Rucphen Family (ERF), the Netherlands Study of Depression and Anxiety (NESDA), the Netherlands Twin Registry (NTR), and the Rotterdam study	AGES-RS: SNPs were excluded with call rates <97%, MAF of <1%, HWE *p* value <10^−6^, and low imputation quality (R^2^ <0.3) ERF: SNPs were excluded with call rates <95%, MAF of <1%, and R^2^<0.3 NESDA and NTR: SNPs were excluded with call rates <95%, MAF of <1%, and R^2^ <0.3	ICHD, 2nd edition for all population-based studies except AGES-RS (self- reported headache)	rs9908234 (*NGFR*), rs11636768 (*AGBL1*), rs10275320 (*MACC1*), rs4939879 (*LIPG*), rs4861775 (*AGA*), rs986222 (*KIF20B*), rs6107848 (*BMP2*), rs140174 (*IGLL1*), rs1146161 (*TSPAN2*), rs4742323 (*KDM4C*) Note: This study selected the SNPs with the significant association with p-value ≤ 5 × 10^−5^.
Chasman et al. (PubMed ID: 21666692) (Chasman et al. 2011)	5,122 migraine, 1,826 migraine without aura, 1,177 migraine with aura, 18,108 controls	American women with European ancestry, Women’s Genome Health Study (WGHS): self-reported migraine Genetic Epidemiology of Migraine’ (GEM): IHS, 1988 * Study of Health in Pomerania’ (SHIP): IHS, 2004* International Headache Genetics Consortium (IHGC): ICHD-II criteria	Samples were excluded for having a genotyping rate of <90%, MAF of <1% SNPs were excluded with call rates <86%, and MAF of <1%		PRDM16, TRPM8, SEPT7, C8orf79, LRP1. Note: This study selected the SNPs with the significant association with p-value ≤ 5 × 10^−6^.
Freilinger et al. (PubMed ID: 22683712) (Freilinger et al. 2012)	2,326 migraine without aura, 4,580 controls,	European descent, including the German dataset (1,208 cases of migraine without aura and 2,564 controls) and the Dutch dataset (1,118 cases of migraine without aura and 2,016 controls)	Samples were excluded with MAF of <1% and HWE *p* value <10^−6^ SNPs were excluded for having a call rate <97%, genotyping rate of 97% with cryptic relatedness, noncryptic relatedness of π_hat >15.0%	ICHD, 2nd edition and the self-reported cases for the clinic-based Leiden University Migraine Neuro Analysis (LUMINA) study website	rs1050316 (*MEF2D*), rs3790455 (*MEF2D*), rs10733092 (*HFM1*), rs17350991 (*RABGAP1L*), rs6756590 (*MARCH4*), rs7640543 (*TGFBR2*), rs12641989 (*RGS12*), rs9381462 (*PHACTR1*), rs1332847 (*PHACTR1*), rs9349379 (*PHACTR1*), rs2327621 (*PHACTR1*), rs7739181 (*PHACTR1*), rs2499797 (*FHL5*), rs11757063 (*FHL5*), rs11777116 (*ADAM28*), rs6478241 (*ASTN2*), rs1712517 (*INA*), rs6598163 (*MMP17*) Note: This study selected the SNPs with the significant association with p-value ≤ 5 × 10^−5^.
Anttila et al. (PubMed ID: 23793025) (Anttila et al. 2013)	23,285 migraine, 7,107 migraine without aura, 5,118 migraine with aura, 95,425 controls	European descent, including population-based studies of Avon Longitudinal Study of Parents and Children (British), Australian Twin Migraine, 1958 British Birth Cohort, deCODE Genetics Inc. (Icelandic), Erasmus Rucphen Family (Dutch), Finnish Twin Cohort (Finnish), Nord-Trøndelag Health Study (Norwegian), Netherlands Twin Register (Dutch), Netherlands Study of Depression and Anxiety (Dutch), NFBC1966 (Finnish), Rotterdam Study III (Dutch), Twins UK (British), Women’s Genome Health Study, Young Finns (Finnish); the clinical studies of German migraine without aura, LUMINA migraine without aura, Finnish migraine with aura, German migraine with aura, LUMINA migraine with aura	Samples were excluded with unexpected sex, and call rates <90% SNPs were excluded with call rates <95%, MAF of <1%, 0.30< heterozygosity < 0.35, and HWE *p* value <10^−5^	ICHD, 2nd edition for all populations-based studies except Avon Longitudinal Study of Parents and Children (self-reported), 1958 British Birth Cohort (self-reported), Finnish Twin Cohort (self-reported), NFBC1966 (self-reported)	rs10915437 (near *AJAP1*), rs12134493 (near *TSPAN2*), rs13208321 (*FHL5*), rs4379368 (*c7orf10*), rs10504861 (near *MMP16*), rs2651899 (*PRDM16*), rs2274316 (*MEF2D*), rs7577262 (*TRPM8*), rs6790925 (near TGFBR2), rs9349379 (*PHACTR1*), rs6478241(*ASTN2*), rs11172113 (*LRP1*)
Pickrell et al. (PubMed ID: 27182965) (Pickrell et al. 2016)	53,109 migraine, 230,876 controls	European descent, 23andMe Inc.	Samples were excluded with call rates <98.5% SNPs were excluded with call rates <95%, large allele frequency discrepancies compared to European 1000 Genomes data, HWE *p* value <10^−20^	Self-reported migraine	rs11172113 (*LRP1*), rs2075968 (*PRDM16*), rs9486719 (*FHL5*), rs9349379 (*PHACTR1*), rs1965629 (near *MSL3P1 /TRPM8*), rs140668749 (near *FGF23/FGF6*), rs2274319 (*MEF2D*), rs6081613 (*SLC24A3*), rs12134493 (near *TSPAN2/NGF*), rs1923243 (near *LRRIQ3*), rs149163995 (*ALS2CR8*), rs7220465 (*RNF213*), rs11187838 (*PLCE1*), rs8052831 (near *ZCCHC14/JPH3*), rs4910165 (near *MRVI1/CTR9*), rs1245465 (near NOVA1), rs11624776 (near *ITPK1/MOAP1*), rs61693171 (*C7orf10*), rs3793683 (*INPP5A*), rs10156578 (near *LINGO2*), rs7030607 (*ASTN2*), rs202203062 (*BAZ1B*), rs910187 (*ZMYND8*)
Gormley et al. (PubMed ID: 27322543) (Gormley et al. 2016)	59,674 migraine, 8,348 migraine without aura, 6,332 migraine with aura, 316,078 controls	European descent, including population-based studie of 23andMe Inc., Avon Longitudinal Study of Parents and Children (British), Australian Twin Migraine, 1958 British Birth Cohort, Danish Headache Centre (Danish), deCODE Genetics Inc. (Icelandic), Dutch migraine with aura (Dutch), Dutch migraine without aura (Dutch), Estonian Genome Centre, University of Tartu (Estonian), Finnish migraine with aura (Finnish), German migraine with aura (German), German migraine without aura (German), Health 2000 (Finnish), Nord-Trøndelag Health Study (Norwegian), Northern Finnish Birth Cohort (Finnish), Netherlands Twin Register and the Netherlands Study of Depression and Anxiety (Dutch), Rotterdam Study III (Dutch), Swedish Twin Registry (Swedish), The Tromsø Study (Norwegian), Twins UK (British), Women’s Genome Health Study, Young Finns (Finnish)	SNPs were excluded with high missingness rates (>5%), MAF of <1%, and failing a test of Hardy-Weinberg equilibrium (varied from <10^−20^ to 10^−3^ for all included data source)	ICHD, 2nd edition for all population-based studies except 23andMe Inc. (self-reported), Avon Longitudinal Study of Parents and Children (self-reported), 1958 British Birth Cohort (self-reported), Estonian Genome Centre, University of Tartu (self-reported), Health 2000 (self-reported), Northern Finnish Birth Cohort (self-reported)	rs11172113 (*LRP1/STAT6/SDR9C7*), rs10218452 (*PRDM16*), rs67338227 (*FHL5/UFL1*), rs2078371(near *TSPAN2/NGF*), rs10166942 (*TRPM8*), rs9349379 (*PHACTR1*), rs1925950 (*MEF2D*), rs4814864 (*SLC24A3*), rs1024905 (near *FGF6*), rs186166891 (*C7orf10*), rs10786156 (*PLCE1*), rs10456100 (*KCNK5*), rs6478241 (*ASTN2*), rs4910165 (*MRVI1*), rs12260159 (*HPSE2*), rs77505915 (*CFDP1*), rs17857135 (*RNF213*), rs2506142 (*NRP1*), rs13078967 (near *GPR149*), rs111404218 (near *JAG1*), rs7684253 (near *REST/SPINK2*), rs4081947 (near *ZCCHC14*), rs1268083 (near *HEY2/NCOA7*), rs75213074 (near *WSCD1/NLRP1*), rs28455731 (near *GJA1*), rs6791480 (near *TGFBR2*), rs11624776 (near *ITPK1*), rs6693567 (near *ADAMTSL4/ECM1*), rs144017103 (near *CCM2L/HCK*), rs10895275 (*YAP1*), rs12845494 (near *MED14/USP9X*), rs10155855 (near *DOCK4/IMMP2L*), rs138556413 (CARF), rs1572668 (1p31.1), rs2223089 (*ARMS2/HTRA1*), rs561561 (*IGSF9B*), rs11031122 (MPPED2), rs140002913 (near NOTCH4)
Chen et al. 2017 (PubMed ID: 28952330) (Chen et al. 2018)	1,005 migraine without aura, 1,053 controls	Han Chinese in Taiwan, 85% of patients from a single tertiary medical centre (Taipei Veterans General Hospital), and 15% of patients neurologic clinics	SNPs were excluded for monomorphic in both cases and controls, call rates < 95% in cases and controls combined, MAF of less than 5% and a total call rate of less than 99% in cases and controls combined, and HWE *p* value <10^−8^ in controls	ICHD, 2nd edition	rs10166942 (*TRPM8*), rs1172113 (*LRP1*), rs655484 (*DLG2*), rs3781545 (*GFRA1*), rs10803531 (*GPR39*)

HWE, Hardy-Weinberg equilibrium; ICHD, the International Classification of Headache Disorders; MAF, minor allele frequency; SNP, single-nucleotide polymorphism.

Note: This study selected the SNPs with the significant association with p-value ≤ 5 × 10^−4^.

### Participants in the Taiwan Biobank (the phenotype and outcome datasets)

The Taiwan Biobank (TWB) is a community-based study, which comprises demographic, lifestyle, and health-related outcomes. In our available data, 27,751 recruited individuals were genotyped using customised TWB arrays (TWB 1.0 and TWB 2.0), with GWAS chips able to identify 653,291 (TWB 1.0) and 686,463 (TWB 2.0) SNPs (Chen et al. 2016; Wei et al. 2021). A detailed description of the TWB is available in published literature (Fan et al. 2008; Chen et al. 2016; Wei et al. 2021). In the Taiwan Biobank, structured questionnaires regarding symptoms and signs of migraines are used. Diagnoses of migraines without an aura and those with an aura were applied according to the International Classification of Headache Disorders (ICHD), 2^nd^ version (Society 2004). In Taiwan, the most popular cognitive assessment tool is the traditional Chinese version of the Mini-Mental State Examination (MMSE). Despite being simplified, the traditional Chinese version of the MMSE, for the Han Chinese population in Taiwan, is the most standard and validated tool for physicians to assess cognitive impairment and dementia (Li et al. 2016; Lee et al. 2018). The traditional Chinese version of the MMSE score ranges from 30 (the best) to 0 (the worst) and includes questions on orientation to time (5 points), orientation to place (5 points), registration (3 points), calculation (5 points), memory recall (3 points), language (2 points), repetition (1 point), and complex commands (6 points) (Li et al. 2016; Lee et al. 2018). In the Taiwan Biobank, the MMSE was used by well-trained interviewers, and the score of each corresponding domain was recorded. Of these, 324 participants were assessed using the traditional Chinese version of the MMSE, and none of them had any missing data (Table S1). The mean MMSE was 27.7 ± 2.4, with a range from minimum of 7 and maximum of 30. The present study was approved by the Joint Institutional Review Board of Taipei Medical University (reference no.: N202104112), and written informed consent was obtained from each participant at study recruitment by the Taiwan Biobank.

### Statistical analysis

For strategy 1, identified SNPs were combined into a single IV in the form of a PRS using the following formula:

$$\text{PRS}=\sum_{j=1}^{m}\gamma_{j}G_{j}$$

where G_j_ is the number of effect alleles carried by SNP j and γ_j_ is the regression coefficient between SNPs and migraines. The two-stage least squares (2SLS) method was used to estimate the causal effect on using a single IV—(Islamoska et al. 2020) first, migraine traits were regressed on SNPs to obtain the regression coefficients γ_j_ and the fitted PRS; and (Chuang et al. 2013) second, a causal estimate was obtained using the regression coefficients of cognitive measures (MMSE) on the fitted PRS (up to four decimal places), and this means that the fitted PRS was used as the IV. The instrumental strength of PRS was assessed using the F-statistic, and a value of the F-statistic of < 10 met the general criterion of weak instrument bias (Burgess et al. 2016; CRP CHD Genetics Collaboration 2011). Enrolled participants were categorised into three tertile groups based on the PRS. An analysis of variance (ANOVA) was used to compare continuous variables among the tertile groups, whereas the Chi-squared test was used for discrete variables. The *p* for trend tests of continuous and discrete variables were respectively examined using a generalised linear model regression and the Cochran-Armitage test.

For strategies 2 and 3, inverse-variance weighted (IVW), MR-Egger regression, and weighted median summary MR estimators (β) were employed to validate the causal association between migraines and cognitive impairment. I^2^_GX_ was used to quantify the weak instrument bias for the MR-Egger regression estimator, and an I^2^_GX_ value of < 90% is considered to introduce a relative bias in a causal effect estimate of > 10%. In other words, the MR-Egger regression estimator was invalid and unsuitable if an I^2^_GX_ of < 90% was attained (Bowden et al. 2016; Minelli et al. 2021). In forest plots, associations between SNPs-phenotype (SNPs-migraine, γ_j_) and SNPs-outcome (SNPs-cognition, Γ_j_) for each SNP were expressed. While the beta coefficient (β) was estimated as the ratio of γ_j_/Γ_j_ using the same population in the one-sample MR, β was defined as the ratio of γ_j_/Γ_j_ using two distinct populations in the two-sample MR. An MR funnel plot was used to assess outlier genetic variants, and an outlier SNP was defined as an estimator (β) located over three times the interquartile range for quartile 1 or quartile 3. A leave-one-out sensitivity MR analysis was also performed. The statistical analysis was performed using PLINK software and the ‘mrrobust’ package (Spiller et al. 2019) in STATA software (vers. 17, College station, TX, USA), and statistical significance was defined as a two-tailed *p* of < 0.05.

## Results

### Participants in the Taiwan Biobank

The overall design of the MR analysis is depicted in Figure 1. Initially, 99 SNPs associated with migraines were identified as having genome-wide significance (*p* value < 5 × 10^−8^) and linkage disequilibrium (with *R*^2^ < 0.001 in a window size of 10,000 kilobases and *p* value thresholds for both index and secondary SNPs of < 1 × 10^−5^) in the IEU open GWAS database and in published migraine GWAS research (Anttila et al. 2010; Chasman et al. 2011; Ligthart et al. 2011; Freilinger et al. 2012; Anttila et al. 2013; Gormley et al. 2016; Pickrell et al. 2016; Chen et al. 2018). Of the identified genetic variants of migraines, 18 SNPs in 1386 participants were available in the TWB for further analysis.

### Strategy 1: Instrumental variable analysis with the PRS of migraines

Regression coefficients between the selected 18 genetic variants and migraines are shown (Table S2), and no direct association was observed between the selected genetic variants and cognition as assessed by the MMSE (Table S3). In addition, no significant association was found between the selected genetic variants and confounding factors of age, sex, or educational level (Table S4). The established PRS was significantly associated with traits of migraines (F-statistic = 13.2, *p* = 0.0003).

Characteristics of enrolled participants in the PRS tertile groups are shown in Supplemental Table 1. No significant differences in the characteristics of age, sex, or educational level between the groups were seen. Participants with a higher PRS exhibited a lower MMSE score. In the two-stage least squares regression analysis (Table S5), migraine traits exhibited a causal association with cognitive impairment (global MMSE β = −2.31, 95% confidence interval (CI): −4.56 to −0.06, *p* = 0.0441). PRS models established by migraine traits without an aura (IVs using three SNPs and two SNPs) displayed a high possibility of horizontal pleiotropy (i.e., no direct association between IVs and outcomes) in the causal relationship. Lastly, the sensitivity analysis revealed that PRSs had no significant association in any single MMSE domain (Table S6).

### Strategy 2: Summary level of GWAS data analysis in a single population

In the one-sample MR framework (phenotype and outcome data in the Taiwan Biobank), we performed an association meta-analysis using summary GWAS data, including SNP-phenotype (SNP-migraine, γ_j_) in Table S2 and SNP-outcome (SNP-cognition, Γ_j_) data in Table S3. A meta-analysis of 18 SNPs (Figure S1) demonstrated that migraines were causally associated with cognitive impairment (IVW estimator of β = 2.90; 95% CI: 0.90–4.89; weighted median estimator of β = 5.70, 95% CI: 2.86–8.54; MR-Egger estimator was invalid due to I^2^_GX_ < 90%). Similarly, odds ratios (ORs) were very high (OR = 18.17, 95% CI: 2.46–132.95 for the IVW estimator; OR = 298.86, 95% CI: 17.46–5115.34 for the weighted median estimator) for the association between migraines and cognitive impairment. The MR funnel plot exhibited an outlier, SNP rs9349379 (Figure S2). In the sensitivity analysis with exclusion of the rs9349379 outlier, a meta-analysis of 17 SNPs (Figure S1) still demonstrated a casual association between migraines and cognitive impairment (IVW estimator of β = 2.87; 95% CI: 0.88–4.87; weighted median estimator = 5.38, 95% CI: 2.36–8.41). Equally, the ORs were still very high (OR = 17.63, 95% CI: 2.41–130.32 or the IVW estimator; OR = 217.02, 95% CI: 10.59–4491.76 for the weighted median estimator). Regression plots for IVW and weighted-median estimators are shown (Figure S3). The other sensitivity analysis performed by leaving one genetic variant out revealed consistent results (Figure S4).

### Strategy 3: Summary level of the GWAS data analysis in two distinct populations

In the two-sample MR analysis, we conducted an association analysis using a summary GWAS of SNPs-phenotype (SNPs-migraine, γ_j_) in the IEU open GWAS database (Table S7) and of SNPs-outcome (SNPs-cognition, Γ_j_) in the Taiwan Biobank. A meta-analysis of 18 SNPs (Figure S5) using a two-sample MR analysis showed that migraines were strongly and causally associated with cognitive impairment (IVW estimator of β = 2.43; 95% CI: 1.08–3.78; weighted median estimator of β = 2.19, 95% CI: =0.18–4.20) as well. Equivalently, the corresponding magnitude of risk ratios were very high (OR = 11.35, 95% CI: 2.94–43.81 on the IVW estimator; OR = 8.94, 95% CI: 1.20–66.68). The sensitivity analysis of leaving one genetic variant out revealed that the results were in line with the original estimators of 18 SNPs (Figure S6). The MR funnel plot showed no extreme outliers for the GWAS summary statistics (Figure S7).

## Discussion

Our strategies 1–3 using the one-sample and two-sample MR analyses showed compatible results, and the present study is the first to investigate the causal relationship between migraines and cognitive impairment in a Han Chinese population. In strategy 1, our one-sample analysis using the PRS as a single IV revealed a causal relationship between migraines and a decrease in the global MMSE. In strategy 2, a one-sample MR, analysing β ratios between SNPs-migraine and SNPs-cognition showed a strong causal effect of migraines on cognitive impairment with IVW and weighted-median estimators. In strategy 3, the two-sample MR, using migraine data in the IEU open GWAS database and cognition data in the Taiwan Biobank, confirmed the robust association between migraines and cognitive impairment.

During the last 2 decades, twin and family studies revealed the existence of hereditary factors in migraineurs (Russell and Olesen 1995), and the meta-analysis of twin studies (Polderman et al. 2015) showed 1.5 ∼ 4-fold risks of migraines in first-degree relatives. While a small portion of migraines, such as familial hemiplegic migraines, is explained by the major monogenic effect of a single genetic variant,(Russell and Ducros 2011) in most cases, migraines are considered to be a polygenic disease with multiple genetic variants, which contribute minor effects and lead to disease development (Grangeon et al. 2023). The polygenic nature of migraines justifies the application of an MR approach to investigate causal associations of cognitive impairment. In addition, large GWAS research conducted by Anttila et al. (2013), Freilinger et al. (2012) and Gormley et al. (2016) consistently identified SNPs in the neuronal and vascular regulation pathway which supported the neurovascular nature of common migraine disorders. These mapped loci can help explain that the causal association between migraines and cognitive impairment is through neurovascular pathways.

In strategy 1, the PRS established by 18 or 17 SNPs (with exclusion of an outlier) that were associated with migraines showed consistent results for the association between migraines and decreases in global MMSE scores. None of the single SNPs exhibiting a direct association with the outcome and confounding factors justified the three MR assumptions in the 2SLS analysis. In addition, this supports all of the selected SNPs contributing small effects to migraine disease and fulfils the assumption of the polygenic nature of common migraines. Nonetheless, we discovered the PRS constructed by three or two SNPs that were associated with migraines without an aura exhibited a horizontal pleiotropic effect in causal associations. These three SNPs were respectively mapped to TRPM8, PHACTR1, and MMP16. TRPM8 is expressed in sensory neurons that regulate cold and pain sensations,(Ling et al. 2019) PHACTR1 mediates endothelial inflammation and is a proven risk SNP for multiple vascular diseases of coronary artery disease, hypertension, cervical artery dissection, and fibromuscular diseases,(Gupta et al. 2017) and MMP16 regulates the degradation of extracellular matrix and may play a role in neuropathic pathways and mental disorders (Lakhan and Avramut 2012). We considered this horizontal pleiotropic effect to be caused by the genetic overlap of migraines with cardiovascular diseases (Winsvold et al. 2017) and psychiatric diseases (Bahrami et al. 2022). In addition, a study supporting migraines without an aura demonstrated more-substantial shared genes in acute ischaemic stroke compared to migraines with an aura (Malik et al. 2015).

In strategies 2 and 3, we considered that the IVW estimator was the most precise one to evaluate the causal association in the meta-analysis. On analysing the summary GWAS statistics, in general the estimator of the Egger method was not recommended since it requires the stringent assumption of an extremely low confounding effect (Bowden et al. 2016; Minelli et al. 2021). In our analysis, using the Egger estimator could introduce a substantial bias in interpretation of the causal relationship between migraines and cognitive impairment due to the extremely low values of I^2^_GX_. To robustly confirm the association, we used an estimator of the weighted-median method, which can obtain less-biased results despite only < 50% of SNPs associated with migraines being valid. A recent study (Minelli et al. 2021) simulating the MR analysis on the UK Biobank found that the MR-Egger method could only be safely used with I^2^_GX_ values of > 97% in a one-sample MR, and I^2^_GX_ values of > 91% in a two-sample MR. Consistent with previous studies in Han Chinese populations (Chuang et al. 2013; Liu et al. 2017; Gu et al. 2022; Huang et al. 2022) and a recent meta-analysis study,(Gu et al. 2022) our one-sample and two-sample MR analyses validated a causal relationship between migraines and cognitive impairment. On using IVs, the one-sample and two-sample MR approaches offered methodological novelty of removing confounding factors. This method assisted us in discovering a strong and robust association between migraines and cognitive impairment.

Our study has several strengths. First, we employed an MR approach in the framework of one-sample and two-sample populations to investigate the causal relationship between migraines and cognitive impairment. For example, non-genetic confounding factors may influence the link between migraine and cognition. A secondary analysis from the Rotterdam Study (Wen et al. 2016) indicated that the migraineurs characterised by a more healthier lifestyle showed better cognitive performance than non-migraineurs. Some migraine prophylactic medications, such as anti-epileptic and anti-depressant drugs, have been considered potential causes of cognitive impairment (Sommer et al. 2013; Do and Schnittker 2020). By fulfilling the MR assumptions, our methodological novelty helped remove confounding factors to obtain the true relationship. Second, we applied a rigorous and comprehensive statistical analysis in the PRS analysis and meta-analysis, showed consistent results across all strategies.

However, this study has some limitations. The Taiwan Biobank includes only Han Chinese participants. Most GWAS summary datasets are derived from European populations, such as the UK Biobank (Sudlow et al. 2015; Conroy et al. 2023) and the FinnGen consortium (Kurki et al. 2023), both of which include publicly available data on over 400,000 adults. The Taiwan Biobank (TWB) also contributes significantly, featuring comprehensive phenotype data for the Han Chinese population with a publicly available sample size over 200,000 adults (Wei et al. 2021). The unbalanced sample size of GWAS summary data may reduce the statistical power for ethnicities with smaller sample sizes in two-sample MR (Brown et al. 2016; Shi et al. 2021). This limits the generalisability of our findings beyond Han Chinese. We highlight the necessity of GWAS studies of migraines and cognitive assessments in diverse ethnicities, including South Asian, Greater Middle Eastern, African, Hispanic/Latino, Oceanic, and other ethnic populations.

Few trans-ethnic two-sample MR studies have been published, most focusing on relationships among kidney functions, obesity, and cardiometabolic factors (Kjaergaard et al. 2022; Lee et al. 2022; Wang et al. 2022; Zheng et al. 2022; Wu et al. 2023). This focus is likely because these traits are common quantitative traits, making them easier to access and yielding significant results. These studies (Kjaergaard et al. 2022; Lee et al. 2022; Wang et al. 2022; Zheng et al. 2022; Wu et al. 2023) employed conventional two-sample MR methods and proposed conducting a sensitivity analyses of estimates by IVW, weighted median, or other meta-analysis methods for validation of the results. Our sensitivity analyses supported consistent results (Figure S5). Given the limited availability of large-scale GWAS studies regarding migraine in a Han Chinese population, the true effect size of causal associations is likely underestimated. Only a subset of genetic variants (Table S5) showed significance, reflecting potential differences in genetic architecture between populations of European descent and the Han Chinese population. Despite these limitation, our one- and two-sample MR studies, both within the same population and cross-ethnic, consistently demonstrated a causal relationship between migraines and cognition. Future studies can ensure the reproducibility of this causal association in other ethnicities using the same method as our strategy. Additionally, we used a migraine diagnosis as the criteria of the ICHD, 2^nd^ version rather than the latest ICHD, 3^rd^ version in 2018 (Cephalalgia 2018). All published GWAS literature (Anttila et al. 2010; Ligthart et al. 2011; Freilinger et al. 2012; Anttila et al. 2013; Gormley et al. 2016; Pickrell et al. 2016; Chen et al. 2018) for migraines and the available open GWAS database, however, also used the ICHD, 2^nd^ version.

MR findings enables personalised medicine by identifying genetic predispositions influencing both migraines and cognitive functions. Genetic markers identified through MR can assess the risk of cognitive decline in migraine patients, allowing for early identification and provocative management strategies, including pharmacological (Tzankova et al. 2023) and non-phamacological treatment (La Touche et al. 2023), regular cognitive screening, and timely interventions. For clinical practice, managing migraines, which significantly impact quality of life, often requires the use acute or prophylactic drugs (Tzankova et al. 2023). Migraineurs frequently express concerns that these medications may negatively affect cognition, such as topiramate causing psychomotor slowing (Sommer et al. 2013; Tzankova et al. 2023). Although studies (Thompson et al. 2000; Rorsman and Källen 2001; Lee et al. 2006; Sommer et al. 2013) show these effects are reversible after discontinuation, concerns about the long-term risk of cognitive decline remain. Our study addresses this concern by confirming the biological link between migraines and cognitive impairment, helping to mitigate fears regarding side effects. This is supported by a study (Santangelo et al. 2016) showing that drug-naïve migraine patients exhibited worse global mental performance. Conversely, evidence (Buse et al. 2010; Gil-Gouveia et al. 2016; Gil-Gouveia and Martins 2019) suggests that chronic or frequent migraines have a more prolonged negative impact on cognition compared infrequent migraines.

In conclusion, our analysis of the Taiwan Biobank, including the 2SLS and meta-analysis of summary GWAS statistics in the framework of one-sample and two-sample MR, surmised that migraines could lead to cognitive impairment in ethnic Han Chinese. Future studies can focus on dissecting the shared genetic basis between migraines and cognition function. The inclusion of more diverse ethnic population and comprehensive objective assessments of cognition (e.g., the use of instruments like the trail-making test and the Montreal Cognitive Assessment) can improve the generalisation of study findings. An interdisciplinary approach is essential, with collaborations among physicians, neurologists, geneticists, and psychologists to address both headache symptoms and cognitive health based on MR findings. Informing patients about the implications of MR findings on their health is crucial, increasing adherence to treatment, prediction, and prevention plans.

## Supplementary Material

Supplemental_Tables_0721_track_change.pdf

Supplemental_Figures_0408.pdf

Supplemental_Tables_0721_clean.pdf

graphical_abstract.pdf

## Data Availability

The original data of Taiwan Biobank are available upon providing a formal proposal to the Taiwan Biobank (https://www.twbiobank.org.tw).

## Lists of abbreviations

2SLS: the two-stage least squares
ANOVA: an analysis of variance
GWAS: Genome-wide association study
ICHD: International Classification of Headache Disorders
IV: instrumental variables
IVW: inverse-variance weighted
MMSE: Mini-Mental State Examination
MR: Mendelian randomisation
OR: odds ratio
PRS: polygenic risk score
SNP: single-nucleotide polymorphisms
TWB: The Taiwan Biobank
