# Supplementary material for: Migraines and the association of cognitive impairment: a one- and two-sample mendelian randomization analysis
Source: Dialogues Clin Neurosci. 2026 Mar 9;28(1):107–18. doi: 10.1080/19585969.2026.2636459 (PMC12973854; doi:10.1080/19585969.2026.2636459)
Supplement: Supplemental_Figures_0408.pdf [file TDCN_A_2636459_SM1545.pdf]

Lists of Online Figures.

Figure S1. Meta-analysis of the one-sample Mendelian randomization (MR) association between migraines and cognitive impairment of 18 selected single-nucleotide polymorphisms (SNPs) (A), and of 17 selected SNPs after exclusion of an outlier, rs9349379 (B). The inverse-variance weighted and weighted median estimators were valid and the MR-Egger estimator was invalid due to violation of a weak instrument bias.

Figure S2. Mendelian randomization (MR) funnel plot of the association between migraines and cognitive impairment in one-sample MR on Taiwan Biobank.

Figure S3. Plot of the SNP–outcome (SNP-cognition, Γj) versus SNP–exposure (SNP-migraine, γj) regression coefficients for the one-sample Mendelian randomization analysis on Taiwan Biobank with an inverse-variance weighted (IVW) estimator (A) and a weighted-median estimator (B) while excluding an outlier, rs9349379.

Figure S4. Sensitivity analysis by leaving one genetic variant out for the one-sample Mendelian randomization with an inverse-variance weighted (IVW) estimator (A) and a weighted-median estimator (B).

Figure S5. (A) Meta-analysis of the two-sample Mendelian randomization (MR) association between migraines and cognitive impairment of 18 selected single-nucleotide polymorphisms (SNPs). (B) Plot of the SNP–outcome (SNP-cognition, Γj) versus SNP–exposure (SNP-migraine, γj) regression coefficients for the two-sample Mendelian randomization analysis with an inverse-variance weighted (IVW) estimator (above) and a weighted-median estimator (below).

Figure S6. Sensitivity analysis by leaving one genetic variant out for the two-sample Mendelian randomization with an inverse-variance weighted (IVW) estimator (A) and a weighted-median estimator (B).

Figure S7. Mendelian randomization (MR) funnel plot of the association between migraines and cognitive impairment in two-sample MR.

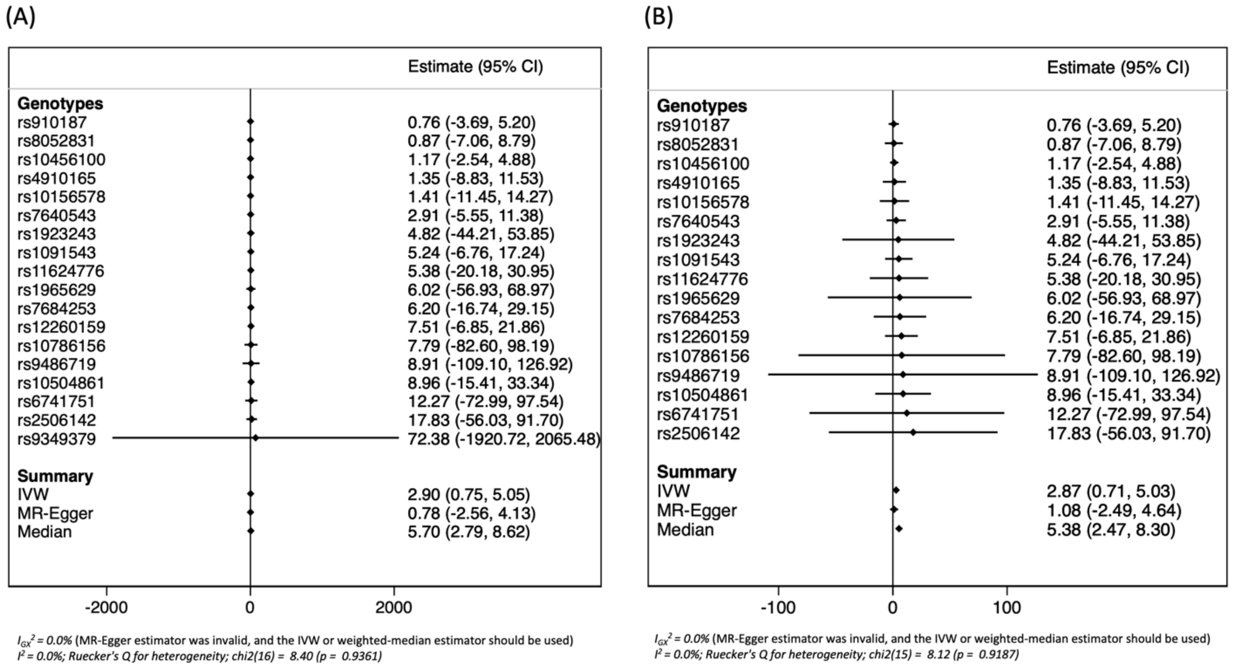

Figure S1. Meta-analysis of the one-sample Mendelian randomization (MR) association between migraines and cognitive impairment of 18 selected single-nucleotide polymorphisms (SNPs) (A), and of 17 selected SNPs after exclusion of an outlier, rs9349379 (B). The inverse-variance weighted and weighted median estimators were valid and the MR-Egger estimator was invalid due to violation of a weak instrument bias.

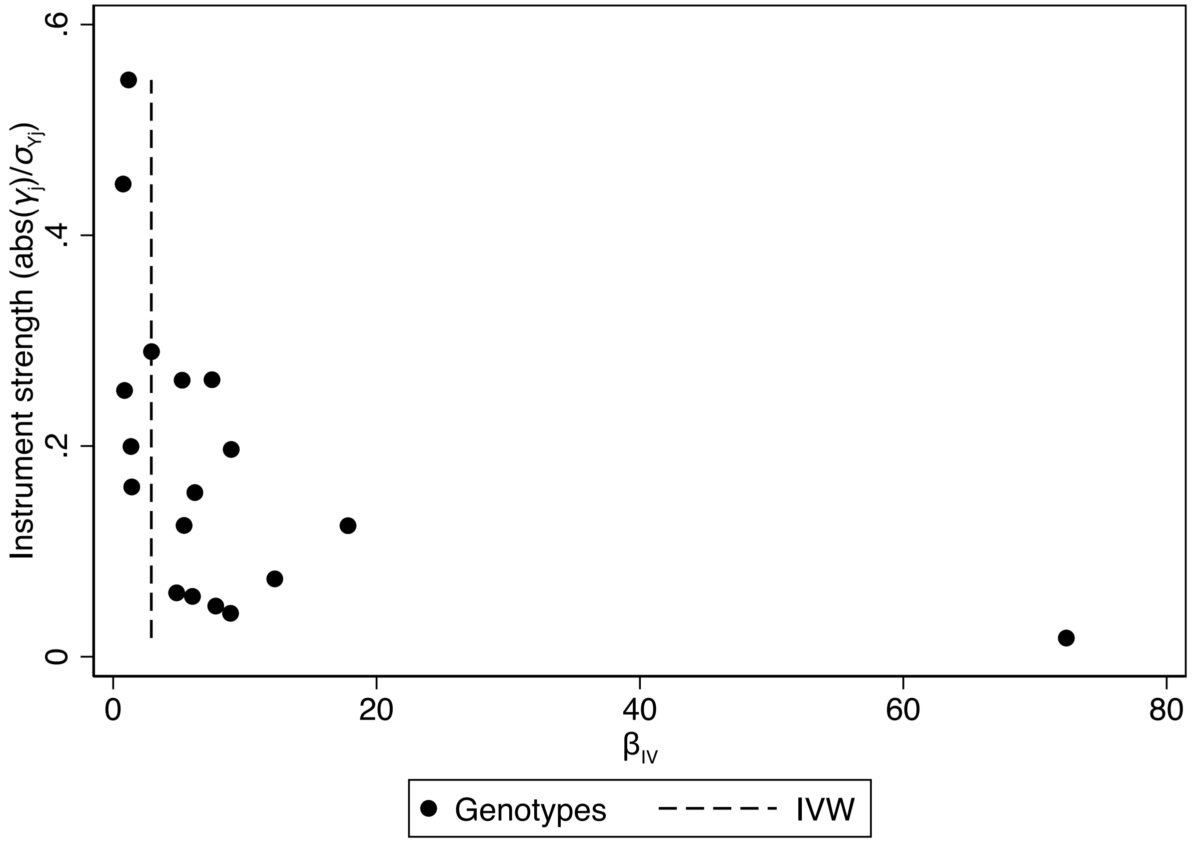

Figure S2. Mendelian randomization (MR) funnel plot of the association between migraines and cognitive impairment in one-sample MR on Taiwan Biobank.

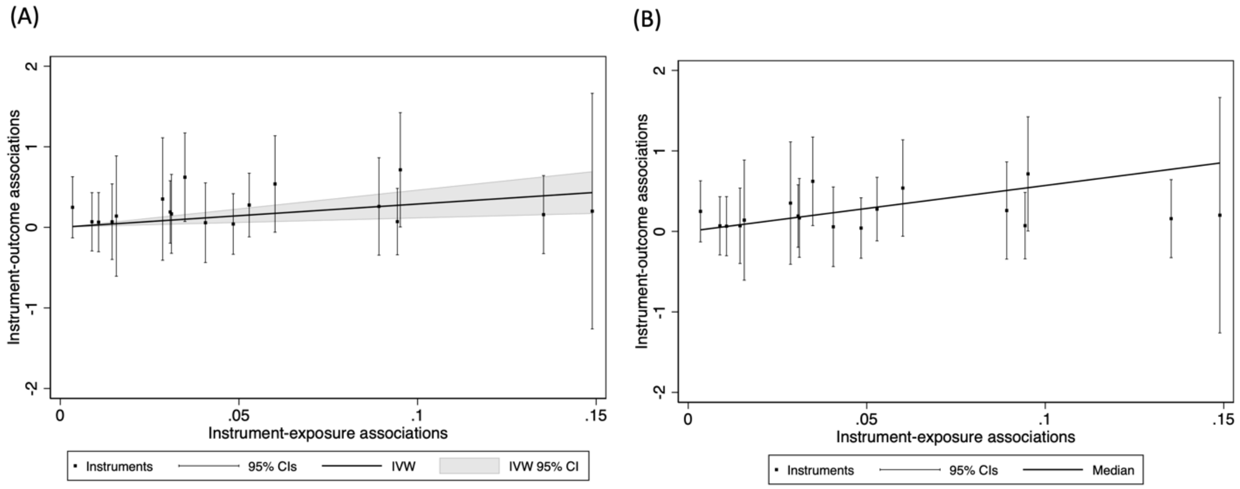

Figure S3. Plot of the SNP–outcome (SNP-cognition, Γj) versus SNP–exposure (SNP-migraine, γj) regression coefficients for the one-sample Mendelian randomization analysis on Taiwan Biobank with an inverse-variance weighted (IVW) estimator (A) and a weighted-median estimator (B) while excluding an outlier, rs9349379.

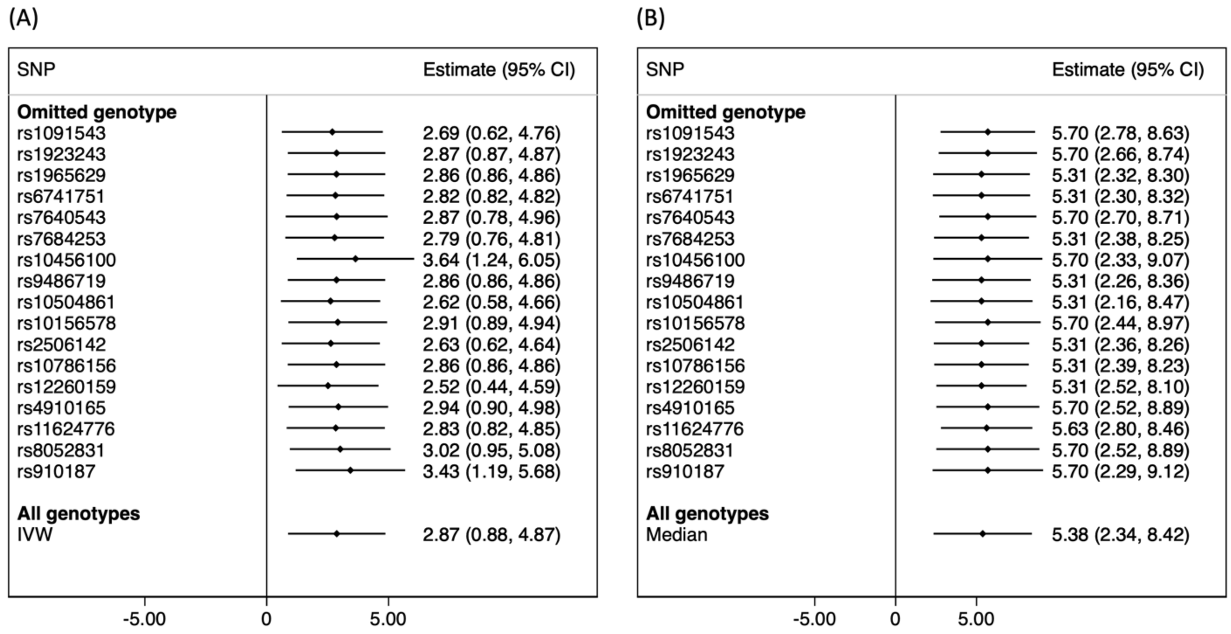

Figure S4. Sensitivity analysis by leaving one genetic variant out for the one-sample Mendelian randomization with an inverse-variance weighted (IVW) estimator (A) and a weighted-median estimator (B).

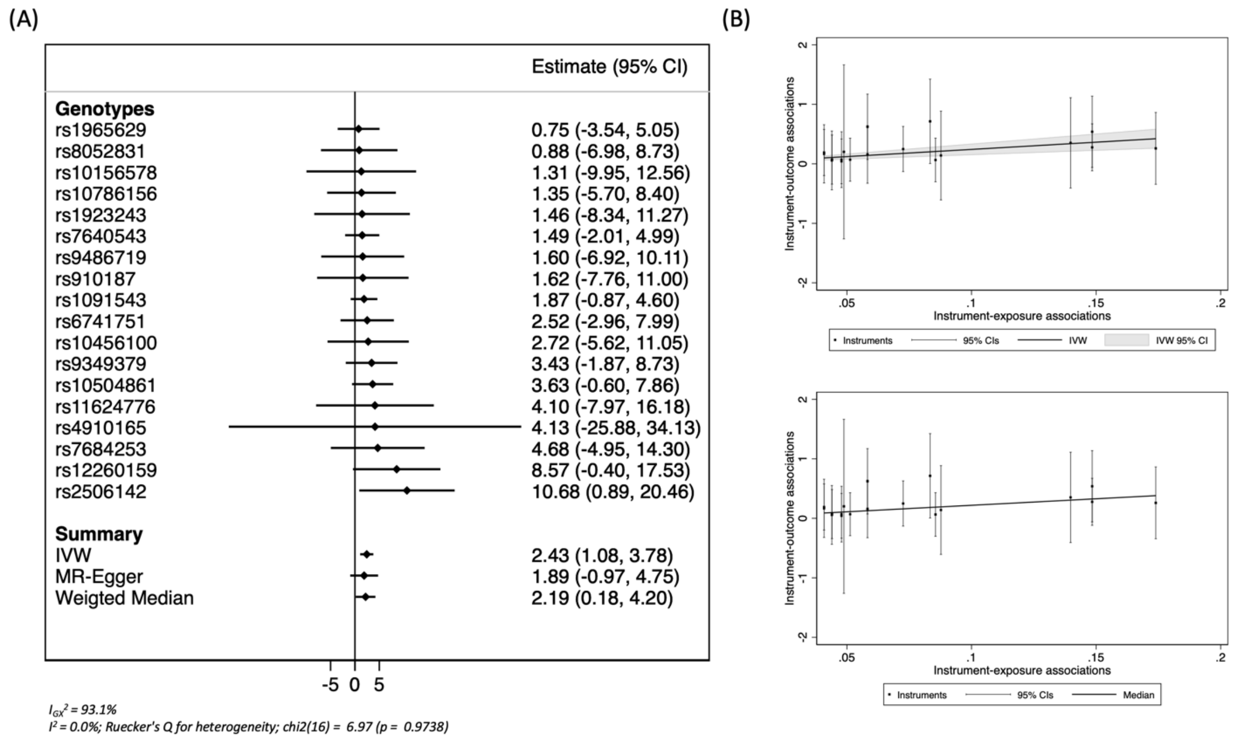

Figure S5. (A) Meta-analysis of the two-sample Mendelian randomization (MR) association between migraines and cognitive impairment of 18 selected single-nucleotide polymorphisms (SNPs). (B) Plot of the SNP–outcome (SNP-cognition, Γj) versus SNP–exposure (SNP-migraine, γj) regression coefficients for the two-sample Mendelian randomization analysis with an inverse-variance weighted (IVW) estimator (above) and a weighted-median estimator (below).

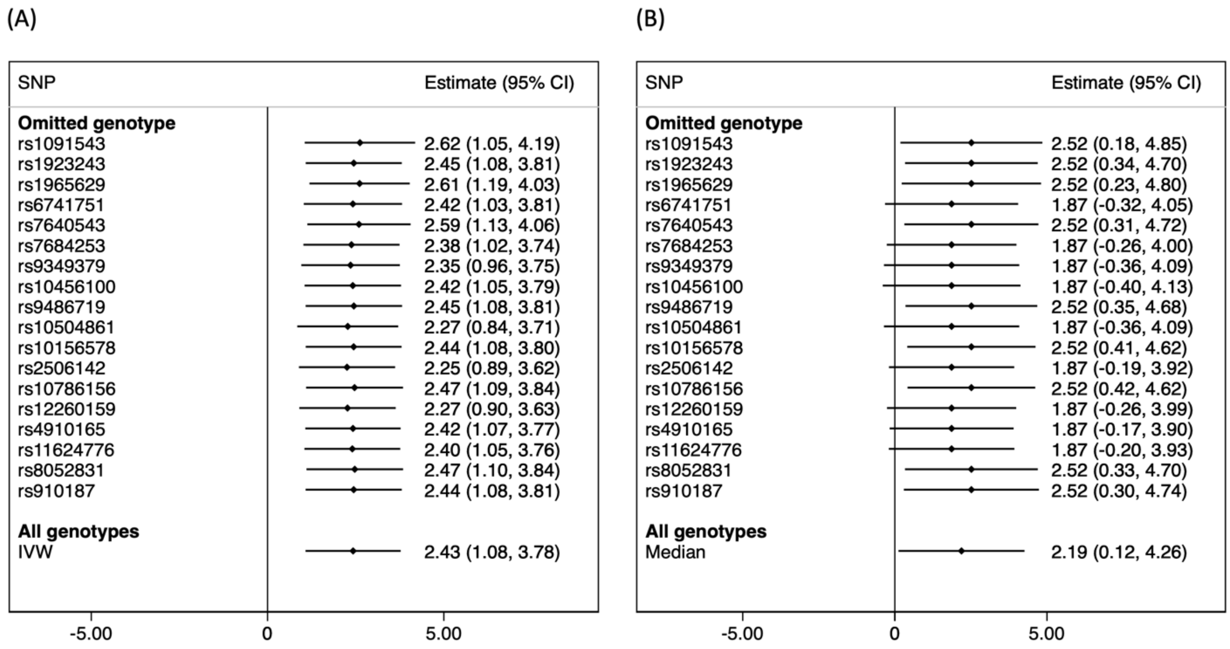

Figure S6. Sensitivity analysis by leaving one genetic variant out for the two-sample Mendelian randomization with an inverse-variance weighted (IVW) estimator (A) and a weighted-median estimator (B).

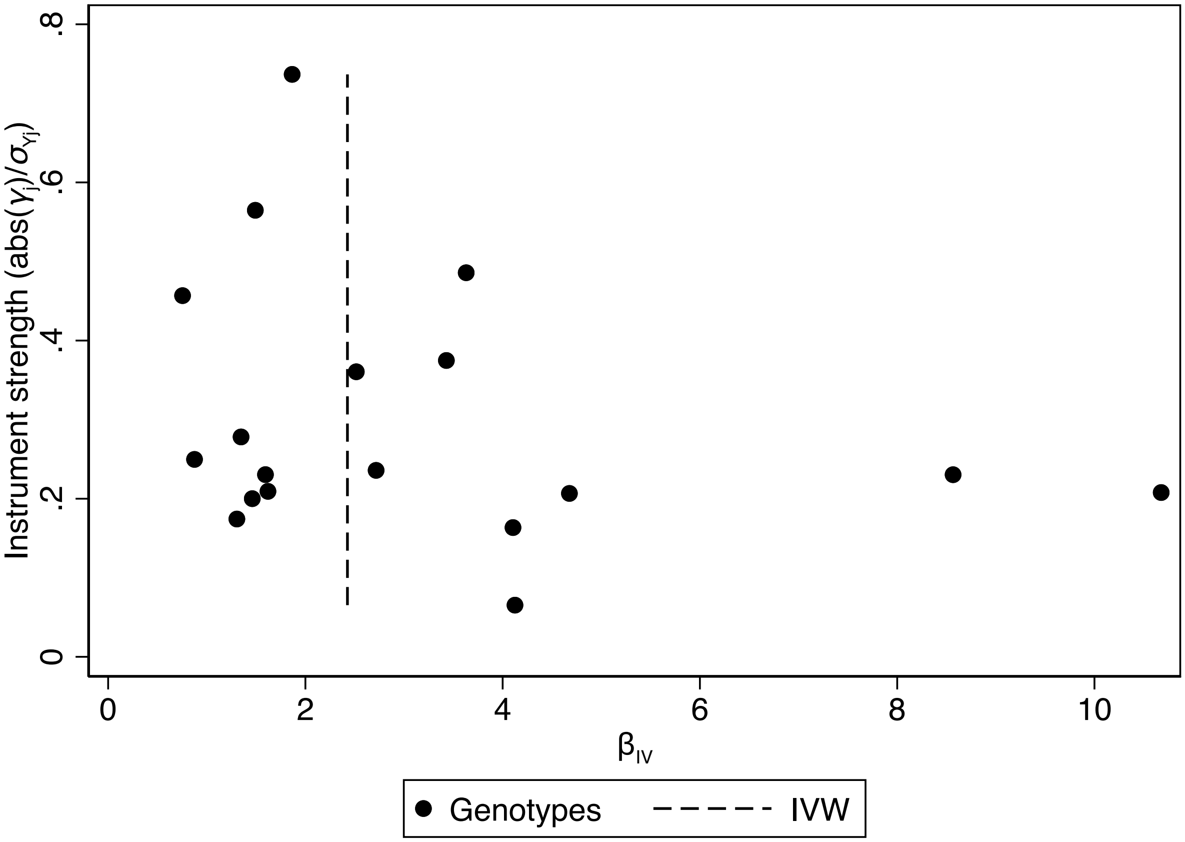

Figure S7. Mendelian randomization (MR) funnel plot of the association between migraines and cognitive impairment in two-sample MR.
