# Supplementary material for: Migraines and the association of cognitive impairment: a one- and two-sample mendelian randomization analysis
Source: Dialogues Clin Neurosci. 2026 Mar 9;28(1):107–18. doi: 10.1080/19585969.2026.2636459 (PMC12973854; doi:10.1080/19585969.2026.2636459)
Supplement: Supplemental_Tables_0721_clean.pdf [file TDCN_A_2636459_SM1544.pdf]

Lists of Online Tables

Table S1. Characteristics of migraine participants according to the tertile levels of the polygenic score

Table S2. Regression coefficients of genetic variants on traits of migraines in the Taiwan Biobank

Table S3. Associations between genetic variants of migraines and cognition (the Mini-Mental Status Examination)

Table S4. *P* values for the associations between migraine genetic variants of migraine and confounders

Table S5. One-sample Mendelian randomization analysis using a two-stage least squares polygenic risk score (PRS) instrument

Table S6. Association between instrumental variable and each domain of Mini-Mental Status Examination

Table S7. Regression coefficients of genetic variants on migraine traits in the IEU open GWAS database

Table S1. Characteristics of migraine participants according to the tertile levels of the polygenic score

PRS	All	Lower tertile	Middle tertile	Upper tertile	*P* value	*P* trend†
N	324	107	109	108		
Range of PRS	0.5226–1.2588	0.5226–0.8621	0.8622–0.9627	0.9628–1.2588	<0.0001	<0.0001
Mean of PRS	0.9130 ± 0.1299	0.7724 ± 0.0642	0.9092 ± 0.0282	1.0560 ± 0.0749	<0.0001	<0.0001
Age	64.6 ± 3.66	64.7 ± 4.0	64.6 ± 3.4	64.3 ± 3.7	0.6870	0.4076
Female sex	256/324 (79.0%)	86/107 (80.4%)	88/109 (80.7%)	82/108 (75.9%)	0.6266	0.8022
Education years	13.1 ± 6.7	13.3 ± 3.2	13.2 ± 3.8	12.8 ± 4.0	0.5212	0.2631
MMSE	27.7 ± 2.4	28.2 ± 1.8	27.4 ± 3.0	27.6 ± 2.3	0.0344*	0.0626
Correlation estimates between PRS and MMSE (β coefficients) ‡	-2.1070	-	-	-	0.0441*	-

Abbreviations: MMSE, Mini-Mental Status Examination*,* *N*, number; PRS, polygenic risk score.

Continuous variables are expressed as mean ± standard deviation.

*Statistical significance at $P < 0.05$.

†The *P* trend was calculated using a generalized linear model for continuous variables and Cochran–Armitage test for discrete variables.

‡The *P* value was calculated using a generalized linear model, with the Mini-Mental State Examination (MMSE) as the dependent variable and the Polygenic Risk Score (PRS) as the independent variable. The predicted equation is $\text{MMSE} = 29.6336 - 2.1070 \times \text{PRS}$.

Table S2. Regression coefficients of genetic variants on traits of migraines in the Taiwan Biobank

SNP	Phenotype	Effect allele	Other alleles	Coefficient (γ_j)	Standard error	*p* value*	EAF	Nearby or in gene	Region	Reference
rs10915437	Migraine	G	A	0.052878607	0.04839590	0.2747	0.34	*AJAP1*	1p36.32	[1]
rs1923243	Migraine	A	C	0.014506630	0.05664021	0.7979	0.22	*LRRIQ3*	1p31.1	[2]
rs1965629	Migraine	G	A	0.010721683	0.04799000	0.8232	0.66	*MSL3P1*	2q37.1	[2]
rs6741751	Migraine without aura	A	G	0.028653151	0.09653044	0.7666	0.12	*TRPM8*	2q37.1	[1]
rs7640543	Migraine	G	A	0.089198036	0.07948972	0.2620	0.09	*TGFBR2*	3p24.1	[3]
rs7684253	Migraine	T	C	0.030777053	0.04857759	0.5265	0.33	*REST*	4q12	[4]
rs9349379	Migraine without aura	G	A	0.003438737	0.04823949	0.9432	0.68	*PHACTR1*	6p24.1	[1-4]
rs10456100	Migraine	T	C	0.135266484	0.05753964	0.0189	0.20	*KCNK5*	6p21.2	[4]
rs9486719	Migraine	A	G	0.015701980	0.09707573	0.8715	0.06	*FHL5*	6q16.1	[2]
rs10504861	Migraine without aura	T	C	0.060108437	0.07610763	0.4298	0.11	*MMP16*	8q21.3	[1]
rs10156578	Migraine	G	C	0.040631380	0.06124268	0.5071	0.17	*LINGO2*	9p21.1	[2]
rs2506142	Migraine	G	A	0.034883938	0.07201639	0.6282	0.11	*NRP1*	10p11.22	[4]
rs10786156	Migraine	C	G	0.008872915	0.04688166	0.8499	0.46	*PLCE1*	10q23.33	[4]
rs12260159	Migraine	G	A	0.095169518	0.07936467	0.2307	0.92	*HPSE2*	10q24.2	[4]
rs4910165	Migraine	G	C	0.148891918	0.14965374	0.3199	0.98	*MRVI1*	11p15.4	[2, 4]
rs11624776	Migraine	A	C	0.031119125	0.05941734	0.6055	0.83	*ITPK1*	14q32.12	[2, 4]
rs8052831	Migraine	A	G	0.048415140	0.04618627	0.2947	0.50	*ZCCHC14*	16q24.2	[2]
rs910187	Migraine	G	A	0.094349145	0.05283666	0.0744	0.76	*ZMYND8*	20q13.12	[2]

Abbreviations: EAF, effective allele frequency; SNP, single-nucleotide polymorphism.

* Statistical significance at $p < 0.0028$ (Bonferroni-based correction).

Table S3. Association between genetic variants of migraines and cognition (the Mini-Mental Status Examination)

SNP	Phenotype	Effect allele	Other allele	Coefficient	Standard error	*P* value*	EAF	Nearby gene	Region
rs10915437	Migraine	G	A	0.27690583	0.2014852	0.2747	0.34	AJAP1	1p36.32
rs1923243	Migraine	A	C	0.06992397	0.2391004	0.7979	0.22	LRRIQ3	1p31.1
rs1965629	Migraine	G	A	0.06456498	0.1872868	0.8232	0.66	MSL3P1	2q37.1
rs6741751	Migraine without aura	A	G	0.35165485	0.3876577	0.7666	0.12	TRPM8	2q37.1
rs7640543	Migraine	G	A	0.25961673	0.3080219	0.2620	0.09	TGFBR2	3p24.1
rs7684253	Migraine	T	C	0.19092932	0.1975168	0.5265	0.33	REST	4q12
rs9349379	Migraine without aura	G	A	0.24889152	0.1936234	0.9432	0.68	PHACTR1	6p24.1
rs10456100	Migraine	T	C	0.15831463	0.2470835	0.0189	0.20	KCNK5	6p21.2
rs9486719	Migraine	A	G	0.13997155	0.3807475	0.8715	0.06	FHL5	6q16.1
rs10504861	Migraine without aura	T	C	0.53880266	0.3055208	0.4298	0.11	MMP16	8q21.3
rs10156578	Migraine	G	C	0.05738702	0.2521625	0.5071	0.17	LINGO2	9p21.1
rs2506142	Migraine	G	A	0.62212486	0.2804130	0.6282	0.11	NRP1	10p11.22
rs10786156	Migraine	C	G	0.06914042	0.1844091	0.8499	0.46	PLCE1	10q23.33
rs12260159	Migraine	G	A	0.71432285	0.3619642	0.2307	0.92	HPSE2	10q24.2
rs4910165	Migraine	G	C	0.20125958	0.7461888	0.3199	0.98	MRVI1	11p15.4
rs11624776	Migraine	A	C	0.16756492	0.2497171	0.6055	0.83	ITPK1	14q32.12
rs8052831	Migraine	A	G	0.04190504	0.1915774	0.2947	0.50	ZCCHC14	16q24.2
rs910187	Migraine	G	A	0.07133559	0.2102953	0.0744	0.76	ZMYND8	20q13.12

Abbreviations: EAF, effective allele frequency; SNP, single-nucleotide polymorphism.

*Statistical significance at *P* value < 0.0028 (Bonferroni-based correction).

Table S4. *P* values for the associations between migraine genetic variants of migraine and confounders

SNP	Phenotype	Age (year)			Sex (female vs male)			Education (year)		
		Coefficient	SE	*P* value	Coefficient	SE	*P* value	Coefficient	SE	*P* value
rs10915437	Migraine	0.11213006	0.38896933	0.7732	0.001068491	0.01578467	0.9460	0.02238209	0.31070285	0.9426
rs1923243	Migraine	0.02103476	0.45558838	0.9632	0.010257344	0.01849704	0.5793	0.29637581	0.37011055	0.4239
rs1965629	Migraine	0.06258466	0.38750807	0.8717	0.010860407	0.01572281	0.4898	0.09868875	0.28864522	0.7326
rs6741751	MO	0.05198620	0.77474354	0.9465	0.011289711	0.03143771	0.7196	0.73622596	0.63424227	0.2466
rs7640543	Migraine	0.71997259	0.63574726	0.2576	0.000730844	0.02577844	0.9774	0.26711149	0.48345190	0.5810
rs7684253	Migraine	0.08927969	0.38836143	0.8182	0.001432950	0.01575983	0.9276	0.12747113	0.30343220	0.6747
rs9349379	MO	0.03136018	0.38701665	0.9354	0.010188465	0.01570305	0.5165	0.33427557	0.30340811	0.2714
rs10456100	Migraine	0.14211133	0.46321543	0.7590	0.039949137	0.01886561	0.0344	0.73099415	0.37361824	0.0513
rs9486719	Migraine	1.38572692	0.78462666	0.0776	0.004850312	0.03187025	0.8791	0.61278539	0.57420142	0.2867
rs10504861	MO	0.17688746	0.60943636	0.7717	0.034197460	0.02471689	0.1667	0.78214138	0.47197786	0.0985
rs10156578	Migraine	0.51475309	0.49139636	0.2950	0.033374646	0.01989857	0.0937	0.16666667	0.39489971	0.6733
rs2506142	Migraine	0.20141192	0.58412175	0.7303	0.011517402	0.02370715	0.6272	0.41222434	0.43382750	0.3427
rs10786156	Migraine	0.14108878	0.37383010	0.7059	0.003629940	0.01517034	0.8109	0.55438689	0.28434181	0.0521
rs12260159	Migraine	0.30506953	0.64679796	0.6372	0.009683697	0.02624761	0.7122	0.05267703	0.55934241	0.9250
rs4910165	Migraine	2.71554022	1.20854677	0.0248	0.029862111	0.04912199	0.5433	0.72101911	1.17600851	0.5402
rs11624776	Migraine	0.82639230	0.47826841	0.0842	0.010678675	0.01942402	0.5826	0.39603960	0.38135624	0.2998
rs8052831	Migraine	0.24754105	0.36771826	0.5009	0.014266399	0.01496003	0.3404	0.29873985	0.29188064	0.3068
rs910187	Migraine	0.78475145	0.42226209	0.0633	0.009170758	0.01715197	0.5929	0.50472695	0.32383780	0.1201

Abbreviations: MO, migraine without aura; SE, standard error; SNP, single-nucleotide polymorphism.

*Statistical significance at *P* value < 0.0028 (Bonferroni-based correction).

Table S5. One-sample Mendelian randomization analysis using a two-stage least squares polygenic risk score (PRS) instrument

Instrumental variable	Instrumental variable with migraine traits			Two-stage least squares analysis	
	Regression coefficient (95% CI)	F-statistic	*p* value	β Coefficient (95% CI)	*p* value
Polygenetic score (18 SNPs)	0.91 (0.42, 1.40)	13.19†	0.0003*	−2.31 (−4.56, −0.06)	0.0441*
Polygenetic score (17 SNPs)‡	0.91 (0.42, 1.40)	13.19†	0.0003*	−2.29 (−4.54, −0.04)	0.0462*
Migraine with aura (15 SNPs)	0.87 (0.37, 1.36)	11.64†	0.0007*	−2.01 (−4.41, 0.39)	0.1004
Migraine without aura (3 SNPs)	1.00 (−1.31, 3.33)	0.72	0.3961	−10.12 (−19.55, −0.71)	0.0352*
Migraine without aura (2 SNPs)‡	1.00 (−1.31, 3.33)	0.72	0.3961	−10.12 (−19.55, −0.71)	0.0352*

Abbreviations: CI, confidence interval; MMSE, Mini-Mental State Examination; SNP, single-nucleotide polymorphism.

* Statistical significance at $p < 0.05$.

† F-statistics of ≥ 10 met the general criterion for avoiding a weak instrument bias.

‡ Instrumental variable analysis of migraine polygenetic score (17 SNPs) excluded an outliner genetic variant rs9349379 from the Mendelian randomization funnel plot (Supplemental Figure 1).

Table S6. Association between instrumental variable and each domain of Mini-Mental Status Examination

MMSE Domain	Two-stage instrumental variable with migraine polygenetic score (18 SNPs)			Two-stage instrumental variable with migraine polygenetic score (17 SNPs)		
	Beta	SE	*P* value	Beta	SE	*P* value
Global score (30 points)	−2.312	1.144	0.0441*	−2.312	1.144	0.0441*
Orientation to time (5 points)	0.021	0.126	0.8700	0.021	0.126	0.8700
Orientation to place (5 points)	−0.285	0.273	0.2974	−0.285	0.273	0.2974
Registration (3 points)	0.027	0.162	0.8676	0.027	0.162	0.8676
Calculation (5 points)	−0.840	0.540	0.1209	−0.840	0.540	0.1209
Memory recall (3 points)	−0.698	0.402	0.0834	−0.698	0.402	0.0834
Language (2 points)	−0.057	0.069	0.4057	−0.057	0.069	0.4057
Repetition (1 point)	−0.129	0.118	0.2782	−0.129	0.118	0.2782
Complex commands (6 points)	−0.351	0.345	0.3111	−0.351	0.345	0.3111

Abbreviations: SE, standard error; MMSE, Mini-Mental State Examination; SNP, single-nucleotide polymorphism.

Table S7. Regression coefficients of genetic variants on trait of migraine in the IEU open GWAS database (European descents)

SNP	Phenotype	Effect allele	Other allele	Regression coefficient	Standard error	*P* value*	EAF	Nearby gene	Region	Ref.
rs10915437	Migraine	G	A	0.148420005	0.026413439	3×10^{-8}*	0.36	AJAP1	1p36.32	[1]
rs1923243	Migraine	A	C	0.047837329	0.007535720	2×10^{-10}*	0.22	LRRIQ3	1p31.1	[2]
rs1965629	Migraine	G	A	0.085557871	0.009362594	7×10^{-19}*	0.66	MSL3P1	2q37.1	[2]
rs6741751	Migraine without aura	A	G	0.139761942	0.017753391	9×10^{-14}*	0.10	TRPM8	2q37.1	[1]
rs7640543	Migraine	G	A	0.173953307	0.027779104	1×10^{-9}*	0.68	TGFBR2	3p24.1	[3]
rs7684253	Migraine	T	C	0.040822027	0.007324007	3×10^{-9}*	0.55	REST	4q12	[4]
rs9349379	Migraine without aura	G	A	0.072570584	0.009537636	6×10^{-22}*	0.62	PHACTR1	6p24.1	[1-4]
rs10456100	Migraine	T	C	0.058268908	0.007254575	7×10^{-13}*	0.28	KCNK5	6p21.2	[4]
rs9486719	Migraine	A	G	0.087738862	0.009362594	6×10^{-21}*	NA	FHL5	6q16.1	[2]
rs10504861	Migraine without aura	T	C	0.148420005	0.026187284	1×10^{-8}*	0.16	MMP16	8q21.3	[1]
rs10156578	Migraine	G	C	0.043951907	0.007324007	2×10^{-8}*	NA	LINGO2	9p21.1	[2]
rs2506142	Migraine	G	A	0.058268908	0.007254575	2×10^{-9}*	0.17	NRP1	10p11.22	[4]
rs10786156	Migraine	C	G	0.051293314	0.004859233	2×10^{-14}*	0.55	PLCE1	10q23.33	[4]
rs12260159	Migraine	G	A	0.083381589	0.014045862	3×10^{-10}*	0.93	HPSE2	10q24.2	[4]
rs4910165	Migraine	G	C	0.048790164	0.008261169	2×10^{-9}*	NA	MRVI1	11p15.4	[2, 4]
rs11624776	Migraine	A	C	0.040822027	0.007324007	8×10^{-9}*	0.69	ITPK1	14q32.12	[2, 4]
rs8052831	Migraine	A	G	0.047837329	0.008021973	2×10^{-9}*	NA	ZCCHC14	16q24.2	[2]
rs910187	Migraine	G	A	0.044016885	0.007812348	4×10^{-8}*	0.63	ZMYND8	20q13.12	[2]

Abbreviations: EAF, effective allele frequency; Ref. references; SNP, single nucleotide polymorphism.

*Statistical significance at *P* value < 0.0028 (Bonferroni-based correction)

\

Reference

1. Anttila V, Winsvold BS, Gormley P, Kurth T, Bettella F, McMahon G, Kallela M, Malik R, de Vries B, Terwindt G *et al*: **Genome-wide meta-analysis identifies new susceptibility loci for migraine**. *Nat Genet* 2013, **45**(8):912-917.
2. Pickrell JK, Berisa T, Liu JZ, Ségurel L, Tung JY, Hinds DA: **Detection and interpretation of shared genetic influences on 42 human traits**. *Nat Genet* 2016, **48**(7):709-717.
3. Freilinger T, Anttila V, de Vries B, Malik R, Kallela M, Terwindt GM, Pozo-Rosich P, Winsvold B, Nyholt DR, van Oosterhout WP *et al*: **Genome-wide association analysis identifies susceptibility loci for migraine without aura**. *Nat Genet* 2012, **44**(7):777-782.
4. Gormley P, Anttila V, Winsvold BS, Palta P, Esko T, Pers TH, Farh KH, Cuenca-Leon E, Muona M, Furlotte NA *et al*: **Meta-analysis of 375,000 individuals identifies 38 susceptibility loci for migraine**. *Nat Genet* 2016, **48**(8):856-866.
