# Supplementary material for: Migraines and the association of cognitive impairment: a one- and two-sample mendelian randomization analysis
Source: Dialogues Clin Neurosci. 2026 Mar 9;28(1):107–18. doi: 10.1080/19585969.2026.2636459 (PMC12973854; doi:10.1080/19585969.2026.2636459)
Supplement: graphical_abstract.pdf [file TDCN_A_2636459_SM1543.pdf]

Title

Migraines and the Association of Cognitive Impairment: A One- and Two-sample Mendelian Randomization Analysis

Genetic Instrument Variables

GWAS for phenotypes of migraine (with and without aura) from all published studies (99 SNPs)

↓

Genetic instruments passing the criteria of significance level of $p < 5 \times 10^{-8}$ and R^2 measure of linkage disequilibrium < 0.001 (67 SNPs)

↓

Genetic instruments of migraine (with and without aura) identified using the Axiom Genome-wide TWB 1.0 and 2.0 Arrays from Taiwan Biobank (18 SNPs, *N* = 1386)

Three strategies to obtain the causal estimates between migraine and cognitive impairment

(1) Mendelian Randomization

(2) Summary-level Mendelian Randomization

(3) Summary-level Mendelian Randomization

Outcome Datasets

One-Sample MR

Cognitive outcome (Mini-mental status Examination) from Taiwan Biobank

Selected 18 migraine SNP in genes of *AJAP1, LRRIQ3, MSL3P1, TRPM8, TGFBR2, REST, PHACTR1, KCNK5, FHL5, MMP16, LINGO2, NRP1, PLCE1, HPSE2, MRVI1, ITPK1, ZCCHC14, ZMYND8*

↓

(1) Polygenic risk scoring method (β = −2.31, 95% CI: −4.56 to −0.06)
(2) Associate estimates by IVW an weighted median methods (β = 2.90; 95% CI: 0.90–4.89) (β = 5.38, 95% CI: 2.36–8.41)

Two-Sample MR

Harmonize GWAS data for cognitive impairment from published studies

Selected 18 migraine SNP in genes of *AJAP1, LRRIQ3, MSL3P1, TRPM8, TGFBR2, REST, PHACTR1, KCNK5, FHL5, MMP16, LINGO2, NRP1, PLCE1, HPSE2, MRVI1, ITPK1, ZCCHC14, ZMYND8*

↓

(3) Causal estimates by IVW and weighted median method: (β = 2.43; 95% CI: 1.08–3.78) (β = 2.19; 95% CI: 0.18–4.20)

GWAS, Genome-wide association study; IVW, inverse-variance weighted; MR, mendelian randomization; SNP, single-nucleotide polymorphism.

CH Bai, HY Fan, HA Lin, SF Lin*
